# Microbiological isolates and associated complications of dacryocystitis and canaliculitis in a prominent tertiary ophthalmic teaching hospital in northern China

**DOI:** 10.1186/s12886-024-03323-x

**Published:** 2024-02-05

**Authors:** Xiaobo Tian, Hua Sun, Yanfei Huang, Wenjun Sui, Dan Zhang, Yufeng Sun, Jing Jin, Yueqing He, Xinxin Lu

**Affiliations:** 1 Department of Laboratory Medicine, Beijing Tongren Hospital, Capital Medical University, Beijing, 100176 China; 2 Department of Ophthalmology, Beijing Tongren Hospital, Capital Medical University, Beijing, 100176 China

**Keywords:** Dacryocystitis, Canaliculitis, Microbiologic isolates, Complications, Antimicrobial susceptibility

## Abstract

**Background:**

To report the microbiological isolates, aetiology, complications, antibiotic susceptibilities, and clinical remission of dacryocystitis and canaliculitis in a prominent tertiary ophthalmic teaching and referral hospital located in northern China and to offer appropriate recommendations for preventing and formulating drug treatment strategies.

**Methods:**

This prospective study recruited a total of 477 participants who had been diagnosed with either dacryocystitis or canaliculitis. The cohort comprised 307 patients with chronic dacryocystitis, 111 patients with acute dacryocystitis, and 59 patients with canaliculitis. Purulent discharge from the lacrimal duct was collected using a sterile swab and immediately subjected to microbial culture. Antimicrobial susceptibility testing was conducted following established protocols. All participants were scheduled for follow-up visits within 14 days after receiving antibiotic therapy.

**Results:**

The present findings indicated that women exhibited a higher susceptibility to the condition, as evidenced by the occurrence of 367 cases in comparison to 110 cases among men. Among the 477 patients, definitive causes were established in 59 individuals, accounting for 12.4% of the patients. Additionally, ocular complications were reported by 132 patients, representing 27.7% of the total. Monocular involvement was observed in the majority of cases, with 402 out of 477 patients (84.3%) affected, while binocular involvement was present in 75 patients (15.7%). In total, 506 microbiological strains were recovered from 552 eyes, with *Staphylococcus epidermidis* (16.4%) being the most prevalent microorganism. Other predominant isolates included *Corynebacterium macginleyi* (9.1%), *Staphylococcus aureus* (5.1%), *Streptococcus pneumoniae* (4.9%), Haemophilus (4.4%), *Propionibacterium acnes* (3.5%), and *Eikenella corrodens* (3.1%). Among the 12 isolated fungi, *Candida parapsilosis* accounted for 66.7%. The susceptibility to antimicrobial agents tested in gram-negative bacilli (79.5%) was observed to be higher than that of anaerobic bacteria (76.7%) and gram-positive cocci (55.4%). With pharmacological therapy, the remission rate of acute dacryocystitis (72.7%) was found to be higher than that of canaliculitis (53.3%) and chronic dacryocystitis (42.3%).

**Conclusions:**

This study highlights the microbial spectrum of dacryocystitis and canaliculitis, particularly *C.macginleyi*, *E.corrodens* and *C.parapsilosis*, which are also more frequently isolated. Vancomycin and imipenem may be more effective treatment options. Most cases have an unknown aetiology, and essential preventive measures involve postoperative cleansing of the lacrimal passage following eye and nasal surgeries, as well as the proactive management of rhinitis.

## Background

The presence of microorganisms in cases of dacryocystitis or canaliculitis poses a potential risk for the development of conjunctivitis, keratitis, or endophthalmitis [1]. However, in the field of ophthalmology, eye infections such as conjunctivitis and keratitis are commonly observed, while dacryocystitis and canaliculitis are regarded as less frequent ocular infections [2, 3]. Therefore, there is a dearth of large-scale prospective microbiological studies and timely follow-up of drug therapeutic efficacy. In recent years, the incidence of dacryocystitis and canaliculitis has been more frequently reported in Asia than in Western countries [4–6]. As the foremost and largest tertiary eye teaching hospital in northern China, Beijing Tongren Hospital possesses abundant patient resources and receives a substantial number of referrals from other medical facilities. Consequently, there is an ample supply of cases available for study. The aim of this study was to comprehensively describe the distinctive microbial patterns observed in dacryocystitis and canaliculitis in northern China, with the ultimate goal of offering drug treatment guidance.

Inflammation of the lacrimal duct system encompasses chronic dacryocystitis, acute dacryocystitis, and canaliculitis. The clinical presentation of acute and chronic dacryocystitis includes the reflux of mucoid or mucopurulent secretions. Furthermore, acute dacryocystitis is distinguished by the presence of erythema, oedema, and tenderness [7]. Canaliculitis is diagnosed by the observation of a pouting punctum or purulent discharge in the lacrimal puncta [8, 9]. Currently, the incidence of this condition in adults is approximately 0.02% [10], while in children, it ranges between 5% and 10%, with nearly 95% of affected children exhibiting symptoms at one month old [11, 12]. Neonatal dacryocystitis may occur as a result of developmental abnormalities in the nasolacrimal duct. Historically, eye injuries caused by conjunctivitis, keratitis, or endophthalmitis have received significant attention. However, ophthalmologists and patients often underestimate the severity of dacryocystitis and canaliculitis due to their mild and atypical symptoms. At the initial onset, patients may only exhibit slight epiphora and skin redness, which can lead to a missed diagnosis or misdiagnosis and ultimately result in deterioration. This can have negative impacts on both eye function and aesthetics and can lead to severe complications, such as lacrimal sac cysts and orbital cellulitis. Pathogenic microbes that are not thoroughly cleared can cause ocular surface infection and exogenous endophthalmitis, particularly after glaucoma or cataract surgery, which can ultimately lead to blindness or the need for eyeball enucleation [1, 13, 14]. Shahraki K’s research suggests that timely and appropriate treatment of lacrimal system infection is important to prevent complications such as endophthalmitis [15].

This study examines the microbial isolates linked to acute dacryocystitis, chronic dacryocystitis, and canaliculitis, assesses their antibiotic susceptibility, and investigates their demographic characteristics, aetiology, complications, and clinical remission rates. The objective is to comprehend the epidemiological features of these ailments in the area and offer some appropriate recommendations for preventing and formulating drug treatment strategies.

## Methods

### Participants

From July 2019 to March 2022, patients diagnosed with dacryocystitis or canaliculitis and suspected bacterial or fungal infection in the Ophthalmic Plastic Department of Beijing Tongren Hospital, Beijing, China, were recruited to this prospective study. All patients included in this study were diagnosed with acute dacryocystitis, chronic dacryocystitis, or canaliculitis through standard diagnostic procedures conducted by ophthalmologists.

Acute dacryocystitis can be diagnosed by observing notable reddening, oedema, and the presence of a painful area of induration located just below the anatomical boundary of the medial canthal ligament. Additionally, epiphora and discharge may be observed, and the expression of purulent material through the lacrimal punctum can be elicited by applying pressure to the inflamed tear duct. The diagnosis of chronic dacryocystitis can be established through the presence of epiphora and mucoid discharge, as well as the observation of the occurrence of purulent material reflux upon applying pressure to or irrigation of the lacrimal duct [7]. Symptoms and signs associated with canaliculitis include epiphora, eyelid matting, a swollen, pouting punctum, or purulent discharge [8, 9].

Patients who had taken antibiotics, antimycotics or hormone compounds within 14 days prior to sample collection, as well as those who had undergone previous ocular surgeries such as dacryocystorhinostomy (DCR), lacrimal duct probing, canaliculotomy, or other ocular surgeries, were excluded. Additionally, patients with ocular surface diseases, such as simple conjunctivitis, simple keratitis or simple dry eye, were excluded. Enrolled patients were either first diagnosed and treated in this hospital or referred from other hospitals.

### Sample collection and microbiological culture

Following placement of all patients in the supine position, the area surrounding the eye was aseptically cleaned, and purulent secretion reflux was obtained through the lacrimal punctum for microbiological culture by applying pressure over the lacrimal sac or canaliculus using a sterile cotton swab.

Following collection, it is imperative that the sample be promptly transported to the microbiological laboratory without being subjected to refrigeration. The secretion or pus obtained from the cotton swab was carefully spread onto an agar plate, employing a sterile inoculation ring for a mild three-zone inoculation.

Blood agar, chocolate agar, and MacConkey agar were utilized for the cultivation of aerobic bacteria that required incubation at 37 °C and 5–10% CO_2_ for a minimum of 24 h. Anaerobic blood agar and an anaerobic jar were employed for the cultivation of anaerobic bacteria, necessitating a cultivation period exceeding 72 h. Throughout this duration, the anaerobic jar must not be opened. Sabouraud dextrose agar (SDA) was employed for the isolation and cultivation of fungi, necessitating a cultivation period of no less than 7 days. The growth of fungi was observed on a daily basis. In conjunction with microbial cultivation, the smear samples were observed by microscopy to detect the presence of cellular entities, bacteria, and fungi.

The identification of expanding colonies was accomplished through the utilization of matrix-assisted laser desorption ionization time-of-flight mass spectrometry (MALDI-TOF MS, bruker, Germany). The assessment of antimicrobial susceptibility was conducted in accordance with the guidelines set forth by CLSI (Clinical and Laboratory Standards Institute) and/or EUCAST (European Committee on Antimicrobial Susceptibility Testing) using the disk-diffusion method (Thermo) or the commercially available automated VITEK-2 system (biomerieux, France).

### Data collection and return visits

A comprehensive range of data was collected, comprising demographic information, medical history, symptoms, signs, ophthalmic examinations, imaging examinations, clinical treatment history, complications, prognosis and clinical outcomes, through the use of an electronic medical record system or phone contact. The remission rate of antibiotic therapy within a 14-day period was determined through follow-up procedures.

### Data analysis

Statistical analysis and drawing were performed using SPSS Statistics software (IBM, version 20.0) and GraphPad Prism (version 8.3.0), respectively. Medians and interquartile ranges (IQRs; Q1-Q3) were given for nonnormally distributed data. The chi-square test or Fisher’s exact test was used to compare ratio differences between groups. A *p* value of less than 0.05 was considered to indicate statistical significance.

## Results

### Participant characteristics

In this study, a total of 477 participants were diagnosed with either dacryocystitis or canaliculitis by ophthalmologists, comprising 2.8% of all ophthalmic patients. Among them, 307 patients (64.4%) were diagnosed with chronic dacryocystitis; 111 patients (23.3%), with acute dacryocystitis; and 59 patients (12.3%), with canaliculitis. Additionally, 16 children were classified as having congenital dacryocystitis.

**Fig. 1:**
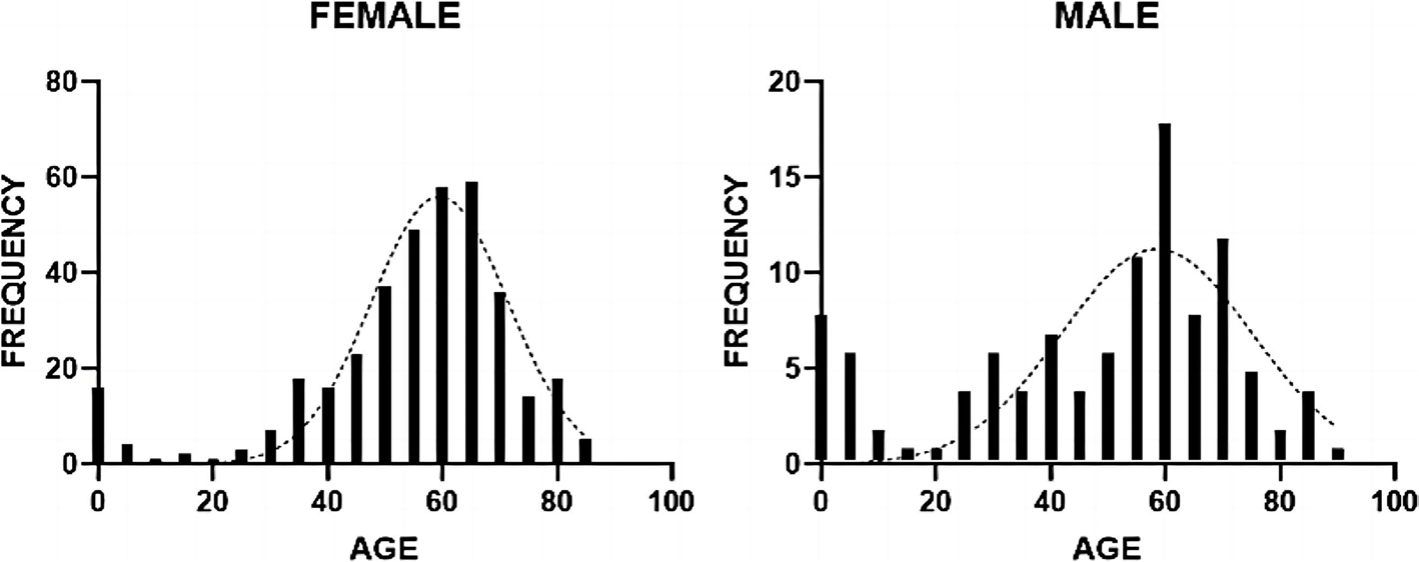
The age distribution of the 367 female patients and 110 male patients

Of the total patients in the study, 402 (84.3%) exhibited monocular involvement, while 75 (15.7%) presented binocular involvement. The female population (*n* = 367) demonstrated a higher prevalence than the male population (*n* = 110). The median age for females was 58 years (IQR, 48, 66; age range, 8 days to 85 years), whereas the median age for males was 55 years (IQR, 31.75, 66; age range, 1 month to 90 years). Notably, the incidence of the condition was higher among children aged 0–10 years and older adults approximately 60 years of age, as depicted in Fig. 1.

### Aetiology and complications

Among the 477 patients included in the study, a comprehensive tracing of aetiology was possible for 59 patients (12.4%). Of these, 45 patients (9.4% of the total patient population) were identified as having ocular or nasal surgery as the cause, while 8 patients had ocular trauma, 5 patients had rhinitis, and 1 patient had trichiasis. The mean duration between the disease onset and the initial doctor visit was 3.5 months, with a range of 8 days to 2 years. Among all patients, 132 individuals (27.7%) experienced complications, with dry eye (59 patients, 12.4% of all patients) and conjunctivitis (37 patients, 7.8% of all patients) being the most prevalent complications. Fifteen patients were diagnosed with lacrimal sac cysts, comprising 13 cases with single complications and 2 cases with multiple complications. Additionally, five patients were diagnosed with orbital cellulitis, consisting of 3 cases with single complications and 2 cases with multiple complications.

### Microbiological isolates

Of the 402 patients with monocular involvement, 272 patients (272 eyes) had positive culture results, and 130 patients had negative culture results. Among the 75 patients (150 eyes) with binocular involvement, 24 patients exhibited negative culture results, 51 patients exhibited at least one positive eye culture, 82 eyes exhibited positive culture results, and 68 eyes exhibited negative culture results. Among the 31 patients with positive binocular culture results, 11 exhibited identical microorganisms, 7 exhibited partially consistent microorganisms, and 13 exhibited completely inconsistent microorganisms.

This study involved the isolation of a total of 506 microorganism strains. The most frequently detected microorganisms were gram-positive cocci (217 isolates, 42.9%), gram-negative cocci (19 isolates, 3.7%), gram-positive bacilli (68 isolates, 13.4%), gram-negative bacilli (129 isolates, 25.5%), anaerobes (61 isolates, 12.1%), and fungi (12 isolates, 2.4%).

**Fig. 2:**
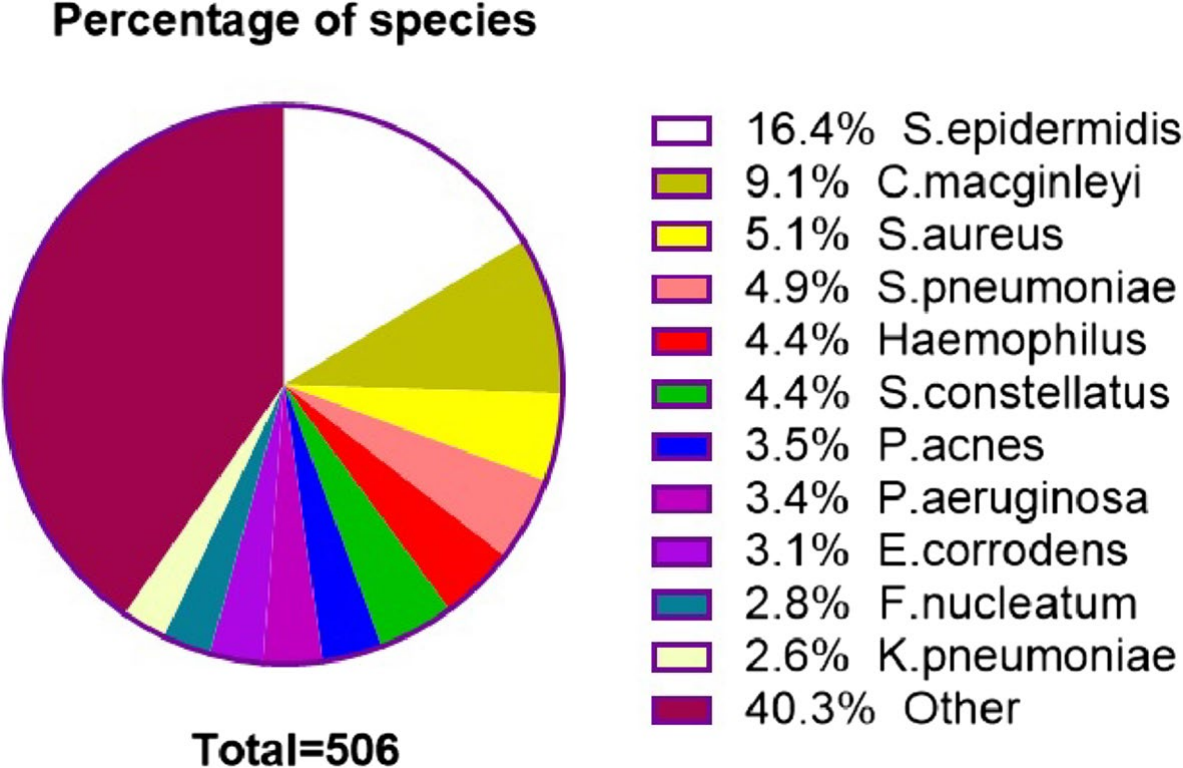
Species and percentage of the main isolated microorganisms

The predominant microorganisms identified in the study are summarized in Fig. 2; Table 1. Additionally, it is noteworthy that *Corynebacterium macginleyi* (46 isolates, 9.1%), *Eikenella corrodens* (16 isolates, 3.1%), and *Fusobacterium nucleatum* (14 isolates, 2.8%) were also isolated in substantial numbers. *Candida parapsilosis* was the most frequently isolated fungus, with a total of 8 strains. Multiple organisms were identified in 27.7%, 18.9% and 21.4% of the patients with chronic dacryocystitis, acute dacryocystitis and canaliculitis, respectively. The statistical analysis of the gram-negative bacterial isolates from the three diseases demonstrated significant differences (*P* = 0.011).

**Fig. 3:**
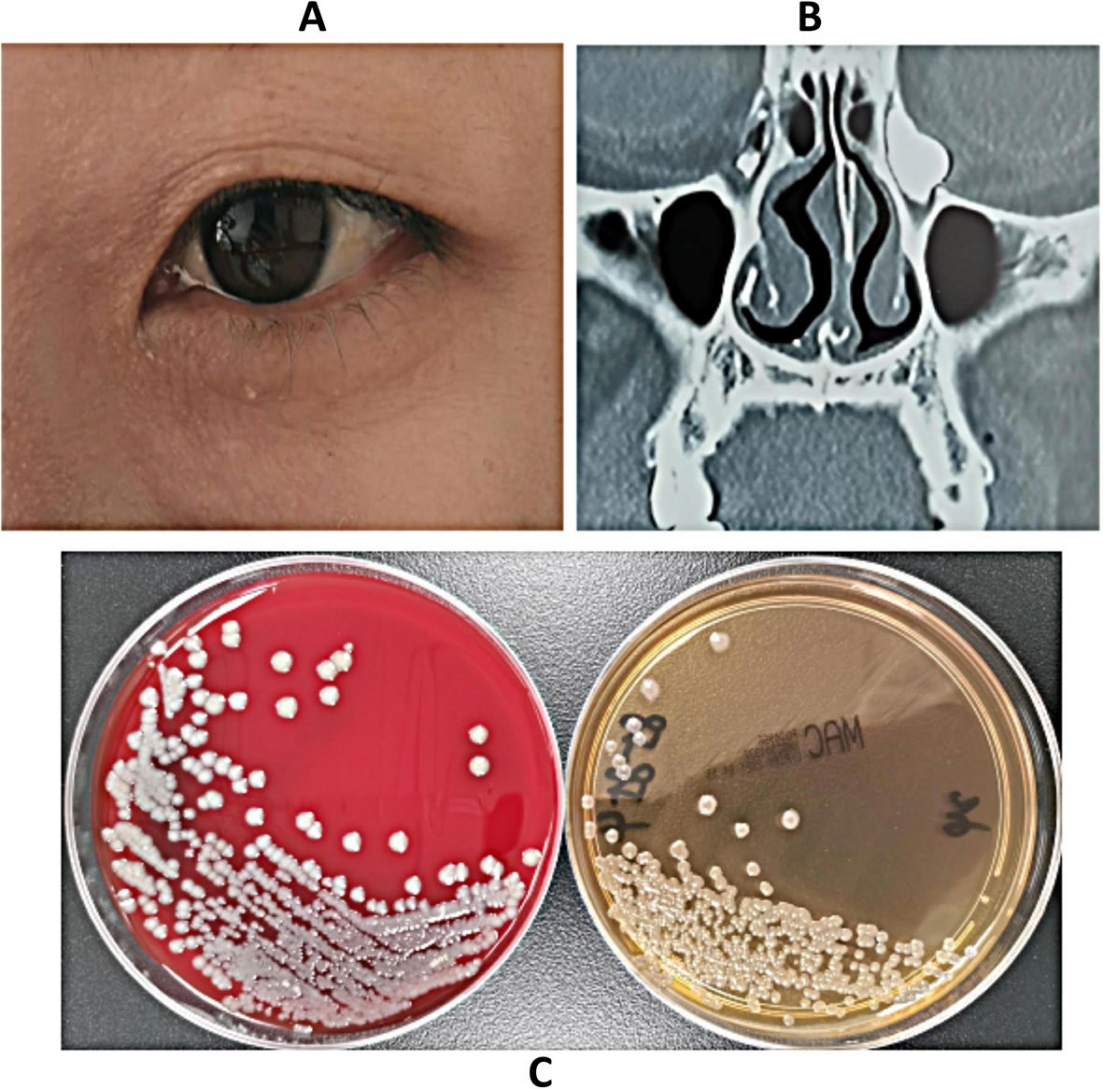
A 31-year-old female patient presented with left dacryocystitis that did not show any improvement for 6 months despite treatment with 0.488% levofloxacin ophthalmic solution. CT imaging revealed the presence of a lacrimal sac cyst. After 24 h, *Stenotrophomonas maltophilia* was cultured from the secretion of puncta lacrimalia

**Fig. 4:**
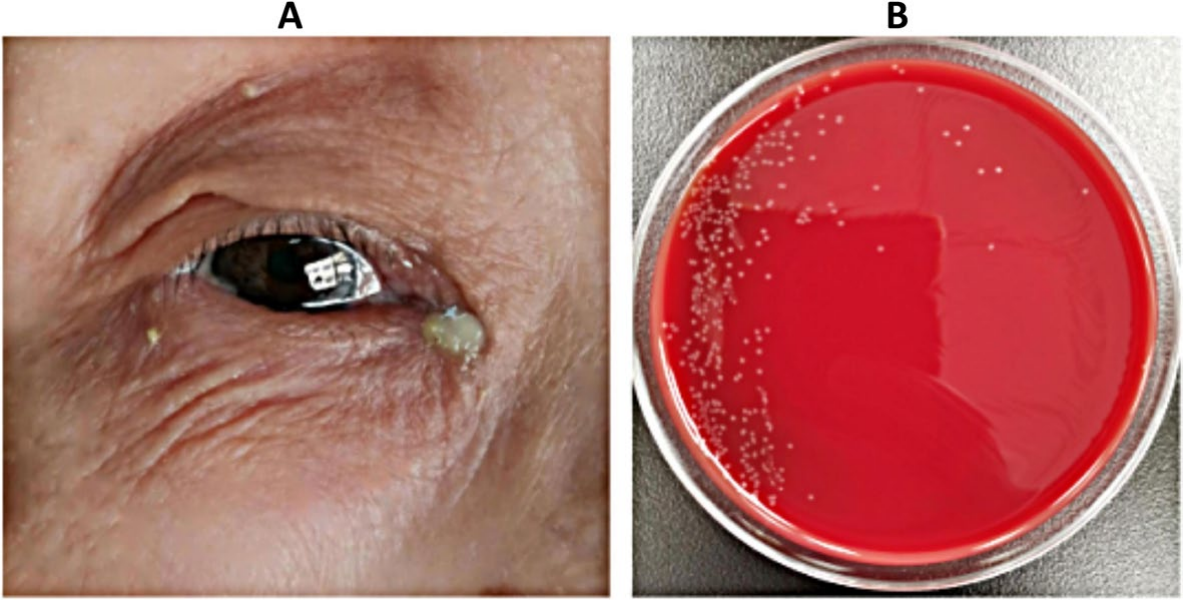
A 78-year-old female patient presented with chronic dacryocystitis caused by *Corynebacterium macginleyi*. There was significant pus at the ocular surface

**Fig. 5:**
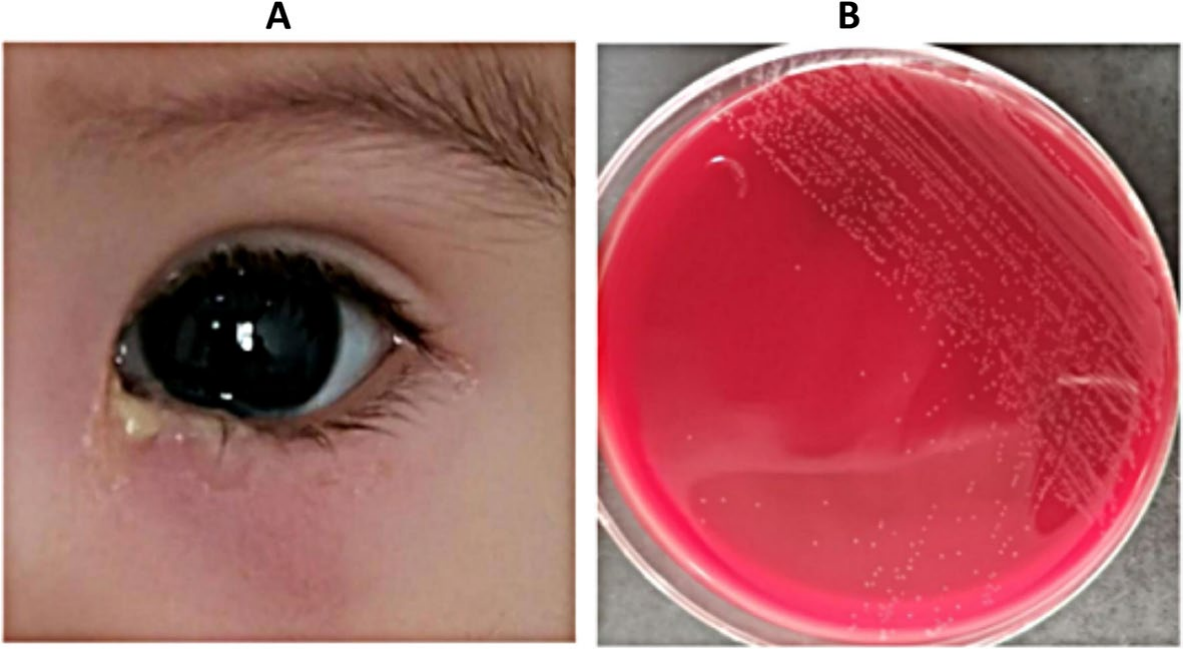
A 7-month-old child presented with chronic dacryocystitis with significant pus caused by *Corynebacterium macginleyi*. There was significant redness and swelling in the area of the left lacrimal sac

**Fig. 6:**
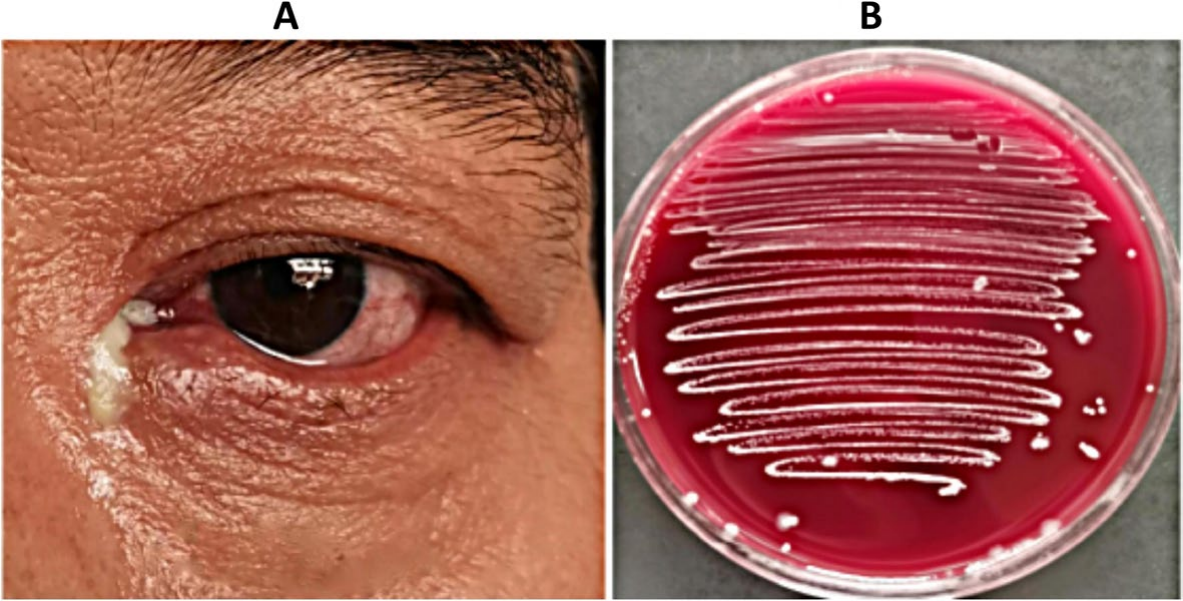
A 52-year-old man presented with acute dacryocystitis with significant pus caused by *Propionibacterium acnes* and accompanied by conjunctivitis

Despite the isolation of only six strains of *Stenotrophomonas maltophilia*, patients exhibited severe symptoms, with one female patient aged 31 years progressing to severe lacrimal sac cyst due to repeated treatment. Notably, this patient did not present with epiphora (Fig. 3). *Corynebacterium macginleyi* is frequently found residing on the ocular surface as either a significant pathogen or a normal flora. The determination of its pathogenicity as being due to either microorganism is contingent upon the evaluation of clinical symptoms. Among patients who have been infected by Corynebacterium macginleyi, as illustrated in Figs. 4 and 5, the presence of a large quantity of purulent discharge in puncta may be considered a primary indicator of pathogenic infection. *Propionibacterium acnes* is the most commonly isolated anaerobic organism, and once it infects the eye, it has a propensity for recurrent invasion (Fig. 6).

**Table 1:** Bacteria and fungi isolated from patients with dacryocystitis or canaliculitis

	Chronic dacryocystitis (Patients = 307)	Acute dacryocystitis (Patients = 111)	Canaliculitis (Patients = 59)	Total (Patients = 477)	*P* value^a^
**Gram-positive coccus (strains)**	**140 (27.7%)**	**48 (9.5%)**	**29 (5.7%)**	**217 (42.9%)**^**b**^	**0.761**
*Streptococcus pneumoniae*	22 (4.3%)	2 (0.4%)	1 (0.2%)		
*Streptococcus constellatus*	12 (2.4%)	5 (1.0%)	5 (1.0%)		
*Streptococcus anginosus*	8 (1.6%)	1 (0.2%)	4 (0.8%)		
*Staphylococcus epidermidis*	52 (10.3%)	23 (4.5%)	8 (1.6%)		
*Staphylococcus aureus*	18 (3.6%)	5 (1.0%)	3 (0.6%)		
Others^c^	28 (5.5%)	12 (2.4%)	8 (1.6%)		
**Gram-negative coccus (strains)** (*Neisseria* or *Moraxella*)	**13 (2.6%)**	**5 (1.0%)**	**1 (0.2%)**	**19 (3.7%)**^**b**^	**0.626**
**Gram-positive bacilli (strains)**	**52 (10.3%)**	**9 (1.8%)**	**7 (1.4%)**	**68 (13.4%)**^**b**^	**0.063**
*Corynebacterium macginleyi*	34 (6.7%)	5 (1.0%)	7 (1.4%)		
Others^d^	18 (3.6%)	4 (0.8%)	0 (0.0%)		
**Gram-negative bacilli (strains)**	**96 (18.9%)**	**24 (4.7%)**	**9 (1.8%)**	**129 (25.5%)**^**b**^	**0.011**
*Klebsiella pneumoniae*	10 (2.0%)	1 (0.2%)	2 (0.4%)		
*Pseudomonas aeruginosa*	12 (2.4%)	5 (1.0%)	0 (0.0%)		
*Stenotrophomonas maltophilia*	3 (0.6%)	4 (0.8%)	0 (0.0%)		
Other^e^	39 (7.6%)	11 (2.2%)	4 (0.8%)		
*Eikenella corrodens*	14 (2.7%)	0 (0.0%)	2 (0.4%)		
*Haemophilus influenzae*	10 (2.0%)	1 (0.2%)	0 (0.0%)		
*Haemophilus parainfluenzae*	8 (1.6%)	2 (0.4%)	1 (0.2%)		
**Anaerobic bacteria (strains)**	**39 (7.7%)**	**10 (2.0%)**	**12 (2.4%)**	**61 (12.1%)**^**b**^	**0.123**
*Propionibacterium acnes*	14 (2.7%)	3 (0.6%)	1 (0.2%)		
*Fusobacterium nucleatum*	10 (2.0%)	1 (0.2%)	3 (0.6%)		
Actinomyces	2 (0.4%)	0 (0.0%)	2 (0.4%)		
Other^f^	13 (2.6%)	6 (1.2%)	6 (1.2%)		
**Fungi (strains)**	**8 (1.6%)**	**4 (0.8%)**	**0 (0.0%)**	**12 (2.4%)**^**b**^	**0.356**
*Candida parapsilosis*	6 (1.2%)	2 (0.4%)	0 (0.0%)		
Other^g^	2 (0.4%)	2 (0.4%)	0 (0.0%)		
**Total (strains)**	**348 (68.8%)**	**100 (19.8%)**	**58 (11.4%)**	**506 (100.0%)**	

^a^All *P* values are derived from chi-square tests, based on the number of patients

^b^The percentage represents the proportion of different bacterial classes but does not represent genera and species

^c^Including 25 strains of Streptococcus, 8 strains of Staphylococcus, 7 strains of Gemella, 3 strains of Enterococcus, 3 strains of Micrococcus, and 2 strains of Kocuria

^d^Including 18 strains of Corynebacterium, 2 strains of Lactobacillus, and 2 strains of Bacillus

^e^Including 25 strains of Enterobacterales, 13 strains of nonfermenters, 1 strain of Pasteurella, 2 strains of *Aggregatibacter aphrophilus* and 13 strains of other fastidious microorganisms

^f^Including 3 strains of other Fusobacterium, 13 strains of Parvimonas, and 9 strains of other anaerobic bacteria

^g^Including *Candida albicans* (1 strain), *Fusarium moniliforme* (1 strain), *Fusarium solani* (1 strain), and *Aspergillus flavus* (1 strain)

### Recheck and return visits

All patients underwent telephone follow-up monitoring. Based on the sensitivity results of antibiotics, a total of 293 patients adhered to the prescribed antibiotic regimen as directed by ophthalmologists. Of these patients, 151 (51.5%) experienced relief from inflammation within 14 days. The remission rate of antibiotic treatment for acute dacryocystitis (72.7%) surpassed that of canaliculitis (53.3%) and chronic dacryocystitis (42.3%). There were significant differences in the three diseases (*P* = 0.002). The most frequently administered antibiotics were quinolones (65.9%), cephalosporins (22.3%), tobramycin (21.2%), and erythromycin (7.3%), with a 16.7% rate of multidrug usage. During the course of drug administration, the treatment of 233 out of 293 patients (79.4%) was solely reliant on the use of eye drops, while in the case of the remaining 60 patients (20.6%), eye drops were used in conjunction with oral or intravenous medication.

Over the course of one year, 74 patients underwent operative intervention, including dacryocystorhinostomy and canaliculotomy. Of these cases, 66 (89.2%) were successfully treated. The cure rate for chronic dacryocystitis (92.2%) was found to be higher than that of canaliculitis (85.7%) and acute dacryocystitis (77.8%). However, statistical analysis revealed no significant differences between the three diseases (*P* = 0.395), as shown in Table 2.

**Table 2:** Remission rate of antibiotic therapy and cure rate of surgery

	Antibiotic		Surgery	
	Return visits (N)	Remission rate(14 days) N(%)	Observation (N)	Cure rate N(%)
**Acute dacryocystitis**	73	53 (72.7)	9	7 (77.8)
**Chronic dacryocystitis**	175	74 (42.3)	51	47 (92.2)
**Canaliculitis**	45	24 (53.3)	14	12 (85.7)
**Total**	293	151 (51.5)	74	66 (89.2)

### Antibiotic susceptibility

 The average susceptibility of a tested drug in 104 strains of gram-negative bacilli was 79.5%. Among these strains, Enterobacterales exhibited the higher susceptibility at 86.9%, while *Pseudomonas aeruginosa* and Acinetobacter showed a susceptibility of 89.7%. Fastidious microorganisms demonstrated a susceptibility of 71.4%, whereas *Stenotrophomonas maltophilia* exhibited a susceptibility of 61.9% (Table 3). Additionally, the average susceptibility of the 180 strains of gram-positive cocci to the tested drug was 55.4%. Among these strains, Staphylococcus exhibited a susceptibility of 48.6%, while Streptococcus showed a susceptibility of 68.4%. Furthermore, the average susceptibility of anaerobic bacteria was found to be 76.7% (Table 4).

**Table 3:** Antibiotic susceptibility of gram-negative bacilli

Enterobacterales (27 strains)		Pseudomonas aeruginosa and Acinetobacter (22 strains)		Fastidious microorganism (48 strains)		Stenotrophomonas maltophilia (7 strains)	
Antibiotic	Susceptibility	Antibiotic	Susceptibility	Antibiotic	Susceptibility	Antibiotic	Susceptibility
PRL	21 (77.8%)	PRL	20 (90.9%)	SXT	28 (58.3%)	MH	6 (85.7%)
TZP	24 (88.9%)	TZP	21 (95.5%)	TZP	40 (83.3%)	LEV	2 (28.6%)
CAZ	23 (85.2%)	CAZ	19 (86.4%)	CAZ	34 (70.8%)	SXT	5 (71.4%)
FEP	24 (88.9%)	FEP	20 (90.9%)	FEP	37 (77.1%)	-	-
IPM	26 (96.3%)	IPM	21 (95.5%)	IPM	41 (85.4%)	-	-
MEM	26 (96.3%)	MEM	21 (95.5%)	MEM	41 (85.4%)	-	-
CIP	24 (88.9%)	CIP	18 (81.8%)	CIP	34 (70.8%)	-	-
LEV	23 (85.2%)	LEV	16 (72.7%)	LEV	33 (68.8%)	-	-
TOB	24 (88.9%)	TOB	21 (95.5%)	MXF	35 (72.9%)	-	-
AMP	19 (70.4%)	AK	21 (95.5%)	AMP	17 (35.4%)	-	-
GAT	24 (88.9%)	GAT	19 (86.4%)	GAT	37 (77.1%)	-	-

*Abbreviations: PRL* Piperacillin, *TZP* Piperacillin/tazobactam, *CAZ* Ceftazidime, *FEP* Cefepime, *IPM *Imipenem, *MEM* Meropenem, *CIP* Ciprofloxacin, *LEV* Levofloxacin, *TOB* Tobramycin, *AMP* Ampicillin, *GAT* Gatifloxacin, *AK* Amikacin, *SXT* Trimethoprim sulfamethoxazole (TMP-SMZ), *MXF* Moxifloxacin, *MH* Minocycline

**Table 4:** Antibiotic susceptibility of gram-positive cocci

Staphylococcus (111 strains)		Streptococcus (69 strains)		Anaerobic bacteria (45 strains)	
Antibiotic	Susceptibility	Antibiotic	Susceptibility	Antibiotic	Susceptibility
PG	8 (7.2%)	P	54 (78.3%)	PG	24 (53.3%)
FOX	20 (18.0%)	E	23 (33.3%)	TZP	42 (93.3%)
E	16 (14.4%)	VA	69 (100.0%)	MEM	40 (88.9%)
VAN	111 (100.0%)	CRO	61 (88.4%)	VA	33 (73.3%)
LEV	52 (46.8%)	LEV	63 (91.3%)	DA	23 (51.1%)
CIP	50 (45.0%)	-	-	MTZ	45 (100.0%)
DA	25 (22.5%)	DA	28 (40.6%)	-	-
GAT	83 (74.8%)	GAT	64 (92.8%)	-	-
MXF	79 (71.2%)	MXF	23 (92.0)	-	-
CN	37 (33.3)	-	-	-	-
SXT	56 (50.5%)	SXT	18 (72.0)	-	-
LZD	111 (100.0%)	LZD	69 (100.0%)	-	-

*Abbreviations: PG *Penicillin G, *FOX *Cefoxitin, *E* Erythromycin, *VAN *Vancomycin, *DA *Clindamycin, *CN *Gentamycin, *LZD *Linezolid, *CRO *Ceftriaxone, *MTZ *Metronidazole

Enterobacterales exhibited favourable susceptibility to the administered drug, with the exception of one strain of carbapenem-resistant *Klebsiella pneumoniae*. Conversely, Staphylococcus demonstrated an inadequate susceptibility to the test drug, with a notable 82.0% of bacterial strains displaying methicillin resistance.

## Discussion

In this study, the patient cohort exhibited notable variations in age and sex, with a higher proportion of females than males. Furthermore, middle-aged and older adults were identified as the population at greater risk. These findings align with prior research conducted in other Asian nations [4, 6]. The underlying reasons for these observations may be attributed to the anatomical structure of the nasolacrimal ducts in women, as well as the widespread use of electronic devices. Among the 477 participants, approximately 18.4% of patients engaged in prolonged computer screen usage for occupational purposes, whereas 14.9% of patients exhibited a keen interest in video content on mobile devices, particularly among the middle-aged and elderly population. Additionally, children between the ages of 1 and 10 years demonstrated a heightened incidence of the condition. The incidence of congenital nasolacrimal duct obstruction (CNLDO) in neonates is approximately 5%, with over 90% of affected children experiencing spontaneous resolution without specific treatment within one year [16]. In cases of severe CNLDO, lacrimal passage irrigation or topical antibiotics may be utilized, although there is no consensus on the optimal timing for intervention [11]. While middle-aged and older women and children are typically the most affected populations, this study revealed that CNLDO can occur in individuals of all ages, ranging from newborn to 90 years old, with the majority of patients experiencing monocular involvement.

Although the incidence of the three diseases was not low, accounting for 2.8% of all eye patients in the past 3 years in this hospital, only 12.4% of patients were able to be aetiologically identified, which typically occurred after ocular and nasal surgery, with rhinitis also being an important pathogenic factor. The average time from symptom onset to medical consultation was 3 months, with patients typically seeking medical attention only when complications arose, including dry eye syndrome and conjunctivitis. Out of the 20 patients diagnosed with lacrimal sac cysts or orbital cellulitis (including single or multiple complications), 17 patients experienced a disease course lasting longer than 2 years. However, 3 patients suffered severe complications within a month. It is noteworthy that although canaliculitis is a rare disease globally and in Asia [2, 8], it accounted for 12.3% of cases in our study population, indicating a high incidence in this region.

The primary challenge that must be addressed pertains to the insufficient utilization of microbial culture in ocular infections by ophthalmologists. A retrospective investigation conducted across seven cities in Eastern China revealed that the microbial culture rate of dacryocystitis was a mere 21.6% [6]. The absence of pathogenic bacteria in empirical therapy is a significant contributor to treatment failure, resulting in high recurrence rates and missed opportunities for optimal antimicrobial therapy. As such, early microbiological diagnosis and antibiotic therapy are crucial. Noninvasive treatment options can alleviate the burden on medical resources and reduce costs for patients, making such therapies desirable for both patients and society.

In this study of dacryocystitis and canaliculitis, we observed a diverse range of microbial communities. *Staphylococcus epidermidis* (16.4%) was the most frequently isolated microbe, alongside *Staphylococcus aureus*, *Streptococcus pneumoniae*, *Haemophilus* spp, *Streptococcus constellatus*, *Propionibacterium acnes*, and *Pseudomonas aeruginosa*, which collectively constituted the dominant microbiome. This finding is consistent with previous studies conducted in other regions of Asia [5, 6, 17–19]. Similarly, the Americas also exhibit a similar microbial spectrum [20]. Similar to *Propionibacterium acnes*, *Staphylococcus epidermidis* is capable of forming biofilms, which allows for prolonged colonization of humans [21] and contributes to the maintenance of skin and mucosal balance [22]. The two bacteria serve as guardians against microbial invasion within the body, primarily through the direct inhibition of pathogen growth due to their competition for nutritional resources with other microorganisms [23]. Additionally, probiotics exhibit inhibitory effects on each other. Research indicates that *Staphylococcus epidermidis* can impede the pathogenicity of *Propionibacterium acnes* [24, 25]. However, it is noteworthy that these two bacteria are also significant ocular pathogens [26, 27]. There is a growing body of evidence indicating that *Staphylococcus epidermidis* may have a pathogenic impact on the human body and serve as a potential microbial source of eye infections, which distinguishes this site from other regions of the human body [28, 29]. It is noteworthy that *Corynebacterium macginleyi* was the second most frequently isolated microorganism in this study (9.1%). This is significantly different from previous studies by other scholars, and approximately 15% of patients infected with this bacterium in this study experienced notable symptoms such as epiphora and pus secretion. In 1995, Riegel provided the initial identification of *Corynebacterium macginleyi* [30], an ocular-specific pathogen that primarily induces bacterial colonization or infection, including keratitis, conjunctivitis, and endophthalmitis [31–34]. Furthermore, the prevalence of *Eikenella corrodens* isolated strains surpasses that of *Haemophilus parainfluenzae* or *Haemophilus influenzae*, a finding that has not been documented in previous investigations [4, 6, 8, 17, 27].

The isolation rate of anaerobic bacteria in the eye was found to be significantly higher than that in other regions of the human body in our study. Brook I’s research conducted in the United States from 1980 to 1990 revealed that Propionibacterium was the second most commonly isolated anaerobic bacteria in cases of dacryocystitis [35]. Similarly, a study conducted by the Rutgers New Jersey Medical School reported that *Propionibacterium acnes* was the third most frequently isolated organism in cases of acute and chronic dacryocystitis [20]. Despite the susceptibility of *Propionibacterium acnes* to most antibiotics, eradication of this bacterium has proven to be challenging, and its toxicity has been previously underestimated [36]. This study revealed that successful recovery of anaerobic bacteria was dependent upon timely inoculation of each sample into anaerobic medium. It is noteworthy to mention that *Fusobacterium nucleatum* ranks second only to *Propionibacterium acnes* in terms of prevalence in this particular study. This finding has not been previously reported in other studies [5, 35, 36]. Conversely, this study demonstrates that *Actinomyces* spp. is not the predominant pathogen responsible for canaliculitis in this specific region, indicating a significant deviation from findings in other regions [37–39].

Although the surgical cure rate for dacryocystitis and canaliculitis is high, research on antibiotic therapy remains insufficient, with only limited reports coming from Asia, Europe, and the Americas [27, 40, 41]. In this study, out of 323 patients with culture-positive results (including 272 with monocular positive cultures and 51 with binocular positive cultures), 293 individuals strictly followed the recommendations for antibiotic treatment based on antimicrobial susceptibility results. The remission rate was over 50% within two weeks, and that for acute dacryocystitis was higher. The present investigation revealed that the gram-negative bacterial strains exhibited greater susceptibility to the tested antibiotics in comparison to their gram-positive counterparts. Additionally, the susceptibility of Streptococcus to the same antibiotics was found to be higher than that of Staphylococcus. Notably, Staphylococcus demonstrated marked resistance to penicillin G and erythromycin, thus warranting a cautious approach towards the empirical use of antibiotics. According to the results of antimicrobial susceptibility tests, it is postulated that vancomycin and imipenem exhibit potential as the most efficacious therapeutic agents for the treatment of gram-positive and gram-negative bacteria, respectively.

The primary approach for addressing ocular surface inflammation is through the use of broad-spectrum topical antibiotics. Historically, fluoroquinolone eye drops have been the preferred topical medication for treating dacryocystitis or canaliculitis [42]. In severe cases, hormone supplementation may be necessary. This investigation revealed that levofloxacin, moxifloxacin, and gatifloxacin had favourable susceptibility against Staphylococcus, Streptococcus, Enterobacteriales, and Pseudomonas, while *Stenotrophomonas maltophilia* exhibited susceptibility rates below 50% [43, 44]. Hence, it is imperative to base treatment decisions on the outcomes of susceptibility tests. Our future research endeavours entail investigating the population fingerprinting of ocular microbes, provided that the microbial culture method is adequate and that the identification of population fingerprinting differences can enhance the understanding of ocular infectious diseases.

## Conclusions

 This study has determined that middle-aged and older adult women, as well as children aged 1 to 10 years, are at a higher risk of morbidity from dacryocystitis or canaliculitis in the northern region of China. The predominant microorganisms isolated from these cases were Staphylococcus, Streptococcus, Haemophilus, and anaerobic bacteria. Significantly, *Corynebacterium macginleyi*, *Eikenella corrodens*, and *Candida parapsilosis* were also more frequently isolated; however, *Actinomyces* spp. were not common. The disease often presented as monocular, and only a few patients had a clear aetiology. The most significant complications were conjunctivitis and dry eye. In the context of prevention, it is imperative to prioritize the maintenance of cleanliness in the lacrimal duct following ocular and nasal surgeries while also providing proactive treatment for rhinitis. Regarding treatment, quinolones may be regarded as the foremost therapeutic choice. Nevertheless, in instances where improvement is not evident, alternative interventions such as vancomycin or imipenem can be considered. Surgical intervention remains a viable course of action if deemed necessary.

## Data Availability

The datasets used and/or analysed during the current study are available from the corresponding author upon reasonable request.
